# Encoded C_4_ homologue enzymes genes function under abiotic stresses in C3 plant

**DOI:** 10.1080/15592324.2022.2115634

**Published:** 2022-09-14

**Authors:** Simin Chen, Wangmenghan Peng, Ebenezer Ottopah Ansah, Fei Xiong, Yunfei Wu

**Affiliations:** Jiangsu Key Laboratory of Crop Genetics and Physiology/Co-Innovation Center for Modern Production Technology of Grain Crops/Joint International Research Laboratory of Agriculture & Agri-Product Safety, Yangzhou University, Yangzhou, China

**Keywords:** C4, C3, abiotic stress, photosynthesis, plant

## Abstract

Plant organisms assimilate CO_2_ through the photosynthetic pathway, which facilitates in the synthesis of sugar for plant development. As environmental elements including water level, CO_2_ concentration, temperature and soil characteristics change, the plants may recruit series of genes to help adapt the hostile environments and challenges. C4 photosynthesis plants are an excellent example of plant evolutionary adaptation to diverse condition. Compared with C3 photosynthesis plants, C4 photosynthesis plants have altered leaf anatomy and new metabolism for CO_2_ capture, with multiple related enzymes such as phosphoenolpyruvate carboxylase (PEPCase), pyruvate orthophosphate dikinase (PPDK), NAD(P)-malic enzyme (NAD(P)-ME), NAD(P) – malate dehydrogenase (NAD(P)-MDH) and carbonic anhydrases (CA), identified to participate in the carbon concentrating mechanism (CCM) pathway. Recently, great achievements about C4 CCM-related genes have been made in the dissection of C3 plant development processes involving various stresses. In this review, we describe the functions of C4 CCM-related homologous genes in carbon and nitrogen metabolism in C3 plants. We further summarize C4 CCM-related homologous genes’ functions in response to stresses in C3 plants. The understanding of C4 CCM-related genes’ function in response to abiotic stress in plant is important to modify the crop plants for climate diversification.

## Background

In C3 photosynthesis, the C3 cycle occurs mainly in mesophyll cells, where carbon dioxide is fixed by Rubisco and then enters into the Calvin cycle. Subsequently, carbohydrates are produced by going through a series of enzymatic chemical reactions.^1^ In contrast to C3 photosynthesis, C4 photosynthesis takes place in mesophyll (M) and bundle sheath (BS) cells in various steps.^2,3^ The distinctions between the biochemical C3 and C4 cycles are associated with (1) the location of photosynthesis occurrence and (2) the mechanism of CO_2_ concentration.^4–6^

The majority of plants performed C3 photosynthesis to synthesize carbon-hydration for life activities, and this process relies on the passive diffusion of CO_2_ from the atmosphere to sites of carboxylation by ribulose-1,5-bisphosphate carboxylase/oxygenase (Rubisco) in chloroplasts.^6^ Compared to C3 photosynthesis reaction, C4 pathway acts carbon concentrating mechanism (CCM) by three key steps: (1) the initial fixation of CO_2_ by phosphoenolpyruvate carboxylase (PEPC) to form a C4 acid, (2) decarboxylation of a C4 acid to release CO_2_ near the site of the Calvin cycle, and (3) regeneration of the primary CO_2_ acceptor phosphoenolpyruvate (PEP) by pyruvate, orthophosphate dikinase (PPDK), which results C4 plant achieve high photosynthetic capacity and higher water and nitrogen use efficiencies.^2,5^

In recent years, there has been widespread interest in the evolutionary diversification of C4 plants. As a convergent phenomenon, C4 photosynthesis is an excellent model for complex trait evolution in response to environmental change. Compared to C3 leaf anatomy, the C4 plants have two important characteristics, Kranz anatomy and less cell number between vascular bundle/tissue.^6,7^ In most C4 plants, Kranz anatomy, which is composed of developed vascular bundle sheath cells and mesophyll cells arranged concentrically around the veins, is rich in chloroplast and proceeds photosynthesis.^8^ C4 plants have higher vein densities which cause the M:BS ratio to be reduced. This allows for greater M-BS contact which facilitates metabolites moving between cells.^7^ In another word, the M/BS (mesophyll cells/bundle sheath cells) ratio of all C4 species is generally low, which reduces the path length and ensures rapid cell-to-cell diffusion of C4 metabolites.^9^ As a consequence, C4 plants are more efficient than C3 plants in photosynthetic product transport and water distribution.^10–12^

In the Oligocene, it was found that the concentration of carbon dioxide gradually decreased, and then reached to modern level by the late Oligocene.^13^ As the CO_2_ concentration reduced, concentration of O_2_ increased, temperatures rise and other environmental elements changed that led plants to recruit series of genes, which enhanced their ability to resist hostile environments by forming some specific mechanisms and structures for survival. Under low CO_2_ concentration, photorespiration enhanced. This likely promoted C4 photosynthesis to evolve.^14^ It is reported that over millions of years, there have been over 60 independent origin events of C4 photosynthesis to enhance their ability to resist hostile environments by forming some specific mechanisms and structures for increasing in energy-costly photorespiration.^15,16^ With adaptive evolution, C4 plants leaves form a special Kranz structure that reduces the distance between the veins, C4 photosynthesis concentrates CO_2_ around the carboxylating enzyme Rubisco by means of PEPCase or other enzymes,^7,17^

Earlier research on tissue structure difference between C3 and C4 plants and the fixing of carbon dioxide during the process of photosynthesis has aroused great interest. Various researches indicate that such factors are key points for studying the evolution of C4 plants to improve the photosynthetic product accumulation rate.^4,6,7,17,18^ It is well known that Rubisco does not only catalyze CO_2_ fixation but also participates in an oxygenase reaction called photorespiration, leading to a reduction in the efficiency of photosynthetic CO_2_ assimilation.^19^ The current research found that the main key enzymes of the C4 pathway include phosphoenolpyruvate carboxylase (PEPCase), pyruvate orthophosphate dikinase (PPDK), which depends on NAD(P) malic enzyme (NAD(P)-ME), dependent on NAD(P) malate dehydrogenase (NAD(P)-MDH) and carbonic anhydrase (CA).^20,21^ Thus, the structure and enzymatic activities of evolved C4 plants enhance their photosynthesis ability and improve photosynthetic substance transport. Compared to C3 plants, C4 photosynthetic enzymes were identified to be important in the resistance of a variety of stresses.

## Function of C4 photosynthetic key genes under abiotic stresses

### Carbonic anhydrases (CAs)

Carbonic anhydrases (CAs), a type of zinc metal enzymes, play essential roles in the conversion of HCO_3_^−^ to CO_2_ in all photosynthetic organisms.^20^ In C4 plant mesophyll, the enzyme converts atmospheric CO_2_ to bicarbonate, which is then used to carboxylate phosphoenolpyruvate (PEP) by the primary carboxylase of C4 plants, PEP carboxylase (PEPC). This reaction initiates the C4 acid transfer cycle that is integral to the carbon concentrating mechanism (CCM) of C4 plants, and leads to CO_2_ concentrations.^22^ Plants have three types of CA: α-, β-, and γ-types involved in CO_2_ uptake, fixation or recycling.^23^ The α-type CA is first found in erythrocytes,^24,25^ whereas the β-type and the γ-type CA were first discovered in plant and archaea, respectively.^26,27^ Some studies have suggested that the main function of carbonic anhydrase may not be to catalyze the mutual conversion of CO_2_ and HCO_3_^−^, but to use this transformation to maintain the required pH level in each compartment of the cell in an animal system.^28^ Then, it can maintain the stability of the intracellular metabolic system. There is a study demonstrating that CAs play a role in the CO_2_ signaling pathway in C3 plants, which regulated the gas-exchange between plants and the atmosphere. In the process of studying *Arabidopsis* mutant plants in the β-carbonic anhydrases, Hu et al. discovered β-CA functioned in guard cells and mediates CO_2_ regulation of stomatal movements.^29^ In leaf mesophyll cells of C4 plants, a cytosolic β-CA catalyzes the first step in C4 photosynthesis. β-CAs are involved in CO_2_ sensing pathway within guard cells and are important in stomatal development. A reaserch of carbonic anhydrase mutants in *Zea mays* displays that CA is connected with stomatal movement in response to CO_2_ and light.^30^ In addition, studies have demonstrated a number of roles attributed to β-CAs found in leaf mesophyll cells, including involvement in refixation of respiratory CO_2_, stress responses, amino acid and lipid biosynthesis, photosystem II activity, and seedling establishment.^20^ Salinity induces an increase in bCA transcript abundance in a maize C4 plant.^31^ It has similar roles in C3 plant as compared with C4 plant. The CAs in Arabidopsis and tobacco have been identified as binding proteins of salicylic acid, which function in an antioxidant role during viral infections.^32,33^ Carbonic anhydrases CA1 and CA4 function in atmospheric CO_2_-modulated disease resistance.^34^ AtCA1 contributes to a negative feedback loop that modulates the plant defense response, which involves in vivo interaction between Turnip mosaic virus (TuMV) HCPro and the Arabidopsis homologue of SABP3.^35^ Cytoplasmic CA2 plays an important role in amino acid biosynthesis and plant growth at low CO_2_ in Arabidopsis.^36^ CA2 over-expressing transgenic plants function in ROS inhibits H_2_O_2_ dependent lignin polymerization, and then caused male sterility.^37^ Under the resistance to the *Plutella xylostella*, AtbCA1 and AtbCA4 protein contents are increased at least a twofold in recombinant lines of Arabidopsis.^38^ Heterologous expression of the rice CA in Arabidopsis leads to enhanced growth on salt medium.^39^ Both salinity and an osmotic stress treatment using polyethylene glycol resulted in an increase in rice seedling total CA enzyme activity, and the level of mRNA coding for a predicted chloroplastic CA isoform.^39^

### Phosphoenolpyruvate carboxylase (PEPCase)

Phosphoenolpyruvate carboxylase is a crucial enzyme that functions in catalyzing phosphoenolpyruvate (PEP) and bicarbonate to form oxaloacetic acid (OAA).^6^ PEPC is found in all plants, green algae, and cyanobacteria, and in most archaea and nonphotosynthetic bacteria, but not in animals or fungi.^40,41^ There are four types of *PEPC* genes in C4 plants: C4 type (green leaf type), root (stem) type, yellow leaf type or C3 type, and bacterial isoform.^42^ Unexpectedly, there are two classes of PEPC in castor oil seeds: class-1 is composed of 107-kDa plant-type PEPC subunits, the other one is formed by the combination of class-1 PEPC and 118-kDa bacterial-type PEPC subunits.^43,44^ Moreover, early studies also have mentioned that PEPCase is probably the most important supplementary product of the citric acid cycle, in addition to the well-known function of fixing CO_2_ in the C4 cycle.^45^ In the C4 photosynthetic pathway, phosphoenolpyruvate carboxylase is one of the rate-limiting enzymes. It is mainly located in the cytoplasm of C4 plant mesophyll cells. The mutants PEPC targeted the cytosolic photosynthetic in *Setaria viridis*, which keep very low PEPC activities, *g*row more slowly than the wild type. Furthermore, the result reveals that the cell wall thickness of mutant leaf reduces and plasmodesmata (PD) density increases at the mesophyll-bundle sheath (M-BS) cell interface.^46^ Other reports also indicate that this enzyme also acts on the regulation of stomatal function, assimilation of nitrogen, stability of pH value and refixation of CO_2_.^47^ In addition to participating in photosynthesis, PEPC also has a variety of physiological functions, including promoting seed germination and formation, regulating guard cells, promoting malate synthesis, providing a respiratory substrate for nitrogen-fixing rhizobia, and playing an important role in plant resistance to abiotic stress.^41,48–50^ *PEPC* genes have been cloned from plants such as *Flaveria, Zea mays, Glycine max* (Linn.) Merr., *Solanum tuberosum* L., *Oryza sativa, Sorghum bicolor*, etc. PEPC plays a crucial role in modulating the balance of carbon and nitrogen metabolism in Arabidopsis leaves.^51^ Bacterial-type PEPC gene fragment concluded that PEPC may function as a metabolic pivot in the regulation of protein and lipid accumulation in Chlamydomonas reinhardtii.^52^

Under drought stress, the photosynthetic rate of transgenic rice with PEPC gene is significantly higher. As a result, the malic acid content increased and stomatal conductance increased, implicating a strong photosynthetic capacity.^53^ C4 *PEPC* genes in C4 plant has stronger adaptability under high temperatures, particularly at low concentration of CO_2_.^54,55^ Transgenic rice plants over-expressing maize C4-*pepc* gene encoding phosphoenolpyruvate carboxylase had higher survival and net photosynthetic rates after 16 d of drought treatment, with higher relative water content in leaves after 2 h of drought treatment.^56^ *ZmPEPC* gene can enhance photochemical and antioxidant enzyme activity, up-regulate the expression of photosynthesis-related genes, delay degradation of chlorophyll, change contents of proline and other metabolites in wheat, and ultimately improve its heat tolerance.^57^ *OsPEPC4* expression reduced line inhibits the assimilation of ammonium, subsequent amino acid synthesis and reduces the level of organic acids. The results show that PEPC plays a key role in the process of ammonium assimilation, which contributes to the growth of rice under NH_4_^+^ stress.^58^ Many studies have shown that C4 type *pepc* genes can improve photosynthetic efficiency and yield potential, when transfered into C3 crops. Heterologous expression of the maize C4-PEPC in rice enhance photorespiratory under low-N levels,^59^ and high maize C4-PEPC transcript levels and activity promote drought tolerance in rice by inducing proline accumulation, enhance photosynthesis, and increase dry weight.^56^

### Pyruvate orthophosphate dikinase, PPDK

PPDK mainly occurs in two sub-chambers, chloroplastic (ChlPPDK) and cytosolic PPDKK (cyPPDKZm1 and cyPPDKZm2), which catalyzes the Pi and ATP-dependent regeneration of PEP from pyruvate and promotes CO_2_ fixation in the C4 pathway.^60^ The chlPPDK is expressed at high levels in the mesophyll cells,^61^ and plays a vital role in C4 photosynthesis, also called C4 PPDK.^62^ Interestingly, *ChlPPDK* and *cyPPDKZm1* in maize are encoded by the same gene controlled by dual promoters with different size of mRNA. At present, the *PPDK* gene has been cloned from some common plants, such as *Zea mays, Oryza sativa, Arabidopsis thaliana, Echinochloa crusgalli*, etc. Same case also exists in C3 Arabidopsis and rice suggesting the gene for chloroplast targeting of PPDK originated before the divergence of C4 plants from their C3 ancestors, as well as between monocots and dicots.^63,64^ *cyPPDKZm1* and *cyPPDKZm2* genes expression level are low in most maize tissues including the root, stem and leaf, but greatly induced in developing seeds.^65–67^ PPDK plays a critical role in the balance between carbon and nitrogen through inorganic pyrophosphate-dependent restriction of ADP-glucose synthesis during endosperm development.^66–68^ The study of a *ppdk* mutant of maize established that the loss of function of this gene caused plant seedlings to die. Also, the sugar signal and nitrogen metabolism of the mutants experienced serious changes, without changes in the anatomical structure of the Kranz.^21^ Arabidopsis PPDK mediates nitrogen remobilization and promotes seed size and seed nitrogen content.^69^ In addition, there are other research results indicating that PPDK enzyme has the ability to mediate the transport of nitrogen from leaves to seeds during the aging process of Arabidopsis, which in turn affects the growth and nitrogen content of seeds.^64^ Rice cytoplasmic PPDK is essential for grain filling, with reduced starch, but increased fat content in the grains.^63^ PPDK also plays an important role when plants face abiotic stresses such as low temperature, drought, and salt stress. PPDK mediated exceptional cold tolerance of carbon assimilation in *Miscanthus* × *giganteus*.^35^ Transgenic rice can significantly reduce the damage caused by drought stress to plant cells, when transgenic PEPC + PPDK double-gene rice was drought treated. Whilst at the same time, the photosynthetic rate and water use efficiency are increased. During this process, the leaf PPDK enzyme activity value and the rate of increase are significantly different from the wild type.^70^

### NADP-malic enzyme (NADP-ME) and NAD-malic enzyme (NAD-ME)

NADP-malic enzyme (NADP-ME) and NAD-malic enzyme are other key enzymes of malate metabolism. The primary function of NADP-ME and NAD-ME is to release CO_2_ from malate, where the CO_2_ released can be refixed by ribulose-1, 5-diphosphate carboxylase/oxygenase (Rubisco). This process is regulated by light and therefore closely related to photosynthesis.^71^ NADP-ME and NAD-ME are two different biochemical subtypes. In NAD-ME plants, aspartate is used as the transport metabolite and malate produced after deamination and reduction, is decarboxylated by NAD-ME in the bundle sheath mitochondria. Whereas in NADP-ME plants, malate is the dominant transport metabolite.^5^ NADP-ME is more efficient under low light conditions, while NAD-ME species have a better photo-adaptive ability. This is seen through the uses of three biochemical subtypes (NADP malic enzyme (NADP-ME), NAD malic enzyme (NAD-ME) and phosphoenolpyruvate carboxykinase (PEP-CK)) of C4 plants as materials, and comparing these plants photosynthetic characteristics under low and high light. In an environment with insufficient light, NADP-ME can better help plant growth.^72^ At present, cDNAs of C4 type *NADP-ME* genes have been cloned in maize, sorghum, and *Flaveria*. NADP-ME is present in the chloroplasts and mitochondria of C4 plant cells. Besides photosynthesis, it participates in a variety of biological processes, including fatty acid synthesis, cytoplasmic pH regulation, and plant defense. The pyruvate produced by the NADP-ME reaction is involved in the synthesis of defensive compounds such as lignin and flavones. Furthermore, NADPH is essential for the stability of the reactive oxygen metabolism system. Therefore, NADP-ME acts on the response of various plants to stress, such as biological stress, drought stress, salt stress, heavy metals, etc.^73^ Some experiments have found that the level of expression of malic enzyme gene (*OsNAD-ME1*, Gene ID: 4343294) in rice changes under salt, alkali, drought, oxidation and other stresses, which indicates that malic enzyme can respond to abiotic stress.^53^ When Hasan et al. studied the NAD-malic enzyme in mesophyll (M) and bundle sheath (BS) cells of *Amaranthus cruentus* L, they demonstrated that a new subtype of NAD-ME (~121 kDa) was formed in the mitochondria of BS cell types, whereas the NAD-ME enzyme activity was noticed to enhance, the subtype disappears after resuming watering under drought conditions. This enzyme contributes to the adaptability of plants in extreme environmental factors.^14^ Additionally, NAD(P)-ME participates in photosynthetic pathway of C4 plants. The carbon dioxide generated by its catalytic decarboxylation reaction, is refixed by Rubisco enzyme and enters the Calvin cycle. There is thus, the potential of breeding high-yield C3 plants with improve photosynthetic rate via cloning and transferring the C4 plant *NAD* (*NADP*)*-ME* gene into C3 crops.

### NAD(P) – malate dehydrogenase

Malate dehydrogenase (MDH) is divided into two types, NAD-dependent and NADP-dependent, whose basic function is to catalyze the biochemical reaction of malic acid and oxaloacetate conversion. MDH is widely distributed in organs including glyoxylates, mitochondria, peroxides, chloroplasts, and cytoplasm.^74^ In plants, it participates in metabolic processes such as C4 cycle, TCA cycle and photosynthesis. Plant MDH is mainly involved in the synthesis and degradation of malic acid, and plays an important role in the cytoplasm and organelle shuttle system: the malate shuttle system closely links different organelle MDHs.^75^ In addition, MDH has also been demonstrated to play an important role in plant response to abiotic stress. Under heavy metal stress conditions, malate dehydrogenase (MDH) in plants catalyzes the conversion of a large amount of oxaloacetate to malate, increasing the malate content. Studies have shown that organic acids such as malic acid can be combined with heavy metals and converted into a nontoxic or less toxic binding state, thereby reducing the toxic effects of heavy metals on plants.^76,77^ Studies have suggested that overexpression of *MDH* gene enhances salt tolerance in plants: malate content increases when plasmid *ZmNADP-MDH* gene is overexpressed in *Arabidopsis thaliana*. And malate salt, which undergoes a series of reactions in the chloroplast and generates through TCA cycle, could promote the increasing of proline synthesis. Consequently, high levels of proline are able to protect plants from salt stress.^78^ The *mMDH2* gene negatively regulates cadmium (Cd) tolerance by modulating reactive oxygen species (ROS) levels and the ROS-mediated signaling, thus, affecting the expression of PDR8.^79^ Loss function of *OsMDH* mutant exhibits a salt stress tolerance phenotype by regulating vitamin B6 content.^80^

## Conclusion

**Figure 1.**
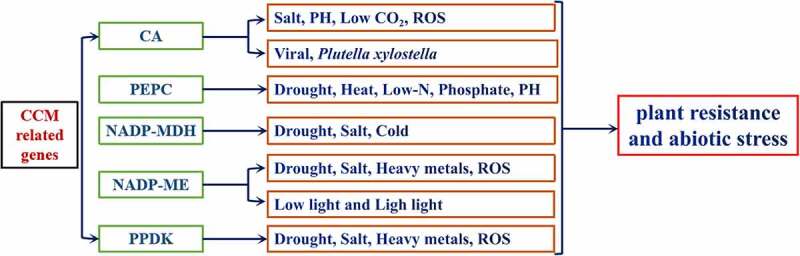
C4-related genes functions in plant development.

C4 plants that evolve from C3 plants are more resistant to unfavorable factors, such as drought, salinity, high temperature, strong light, and so on, among which C4 genes play an important role (Figure 1). Therefore, research on the C4 genes is of great significance as humans are faced with challenges that threaten food security such as the increase in global population, decrease in the area of arable land, global warming, frequent extreme weather, and land desertification. In recent years, someone proposed the C4 rice project,^18^ which is to introduce C4 photosynthetic pathways into C3 plant rice, although the results show that based on the existing veins, the introduction of a small amount of C4 photosynthesis may already be beneficial to the photosynthesis of C4 rice, there are still many problems to be solved. In order to explore the key enzyme functions of the C4 pathway in depth, researchers transferred it into C3 plants for research. ^81^,transferred the maize NADP-ME gene into rice. The research results found that the expression of NADP-ME gene increased in transgenic rice under drought conditions. And the plant’s stomatal conductance decreases, so it has the ability to retain water and drought resistance.^81^ Under drought stress, the photosynthetic rate advantage of PEPC gene transgenic rice is significant, and it is found that its malic acid content rises, the stomatal conductance is higher, and it has a strong photosynthetic capacity.^53^

Current studies have shown that the role of C4 key enzyme gene in plant response to adversity is a complex process in many aspects, especially in drought stress and salt stress. However, some studies have found that it has a significant role in plant response to heavy metal stress, such as cadmium and aluminum. It is not clear. Soil heavy metal pollution has been caused to different degrees, causing plant toxicity, which is caused by the use of organic fertilizers, chemical fertilizers, pesticides, and the discharge of various industrial sewage. An in-depth study of the function of C4 key enzyme gene in response to abiotic stress and its mechanism of action may provide new insights for the study of plant stress tolerance.
